# Multiomic Underpinnings of Drug Targets for Intracranial Aneurysm: Evidence From Diversified Mendelian Randomization

**DOI:** 10.1111/cns.70430

**Published:** 2025-05-10

**Authors:** Yu‐Xiang Fan, Di Lu, Cheng‐Bin Yang, Zi‐Hao Song, Yi‐Guang Chen, Yong‐Jie Ma, Jing‐Wei Li, Hong‐Qi Zhang

**Affiliations:** ^1^ Department of Neurosurgery, Xuanwu Hospital Capital Medical University Beijing China; ^2^ China International Neuroscience Institute (China‐INI), Xuanwu Hospital Capital Medical University Beijing China

**Keywords:** Druggable, eQTL, intracranial aneurysm, Mendelian randomization, phenome‐wide, pQTL

## Abstract

**Aims:**

The absence of pharmaceutics poses challenges in preventing intracranial aneurysm (IA) progression and rupture. This research emphasized identifying drug targets for IA through a druggable genome‐wide Mendelian randomization (MR) analysis.

**Methods:**

A two‐sample MR analysis was performed leveraging *cis‐*expression quantitative trait loci in the blood (*n* = 31,684) and arteries (*n* = 584) aligned with 5883 druggable genes as exposure and the largest IA summary statistics (*n* = 7495) as outcome. Bayesian colocalization analysis, plasma cis‐protein quantitative trait loci (*n* = 35,559), and external IA cohorts (FinnGen, *n* = 2582; Zhou, *n* = 380) were used for validation. A phenome‐wide MR (Phe‐MR) incorporating 783 diseases uncovered side effects. Multivariable MR addressed unmeasured pleiotropy.

**Results:**

Five druggable genes in blood and one in the coronary artery showed significant association with IA risk (*p‐*_*FDR*_ ≤ 0.05). *NT5C2*, *PRCP*, and *CRMP1* shared a common variant with IA (PPH4 ≥ 0.8). The external validation cohorts confirmed the effects of *NT5C2* on IA (FinnGen cohort, Odds Ratio [OR], 0.81, 95% Confidential Interval [95% CI] 95% CI, 0.707–0.930; *p* = 0.003; Zhou cohort, OR, 0.68, 95% CI, 0.469–0.983; *p* = 0.041). The genetically predicted protein level of *PRCP* validated an inverse association with IA risk (OR, 0.734; 95% CI, 0.561–0.959; *p =* 0.023). The Phe‐MR revealed insignificance for *NT5C2* or *PRCP*. Direct causal effects on IA were 0.60 (95% CI, 0.457–0.797; *p* = 1.36E‐05) for *PRCP* and 0.67 (95% CI, 0.527–0.860; *p* = 0.002) for *NT5C2* after adjusting for IA modifiable risk factors.

**Conclusions:**

*NT5C2* and *PRCP* were identified as potential drug targets for IA, with effects independent of known modifiable risk factors. Targeting *NT5C2* and *PRCP* appeared exclusively effective and safe.

## Graphical abstract

*NT5C2* and *PRCP* were identified as druggable targets for intracranial aneurysms (IA) with multiomic druggable genome‐wide Mendelian randomization, colocalization analysis, and external validation support. The causal effect of *NT5C2* and *PRCP* on IA was independent of the modifiable IA risk factors, such as high blood pressure, smoking, and insomnia. Targeting *NT5C2* and *PRCP* appeared exclusively effective and safe for IA, including ischemic stroke and hypertension, which warranted future investigations.

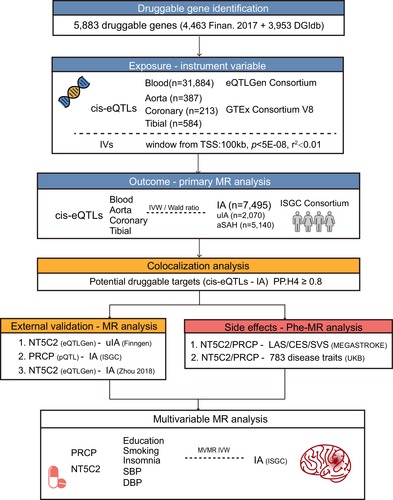

**Abbreviations**
aSAH: Aneurysmal subarachnoid hemorrhage
CES: Cardioembolic stroke
cis‐eQTLs: Cis‐expression quantitative trait loci
cN‐II: Cytosolic 5′‐nucleotidase ii
DBP: Diastolic blood pressure
DGIdb: Drug–gene interaction database
FDR: False discovery rate
GTEx: Genotype‐tissue expression project
IA: Intracranial aneurysms
ISGC: International stroke genetics consortium
IVs: Instrument variables
IVW: Inverse variance weighted
LAS: Large artery atherosclerosis
MR: Mendelian randomization
OR: Odds ratios
Phe‐MR: Phenome‐wide Mendelian randomization
PPH4: Posterior probability of hypothesis 4
PPI: Protein–protein interaction
PRCP: Prolyl carboxypeptidase
RCT: Randomized clinical trial
SAIGE: Scalable and accurate implementation of generalized mixed model
SBP: Systolic blood pressure
SNPs: Single‐nucleotide polymorphisms
SVS: Small vessel stroke
TSMR: Two‐sample Mendelian randomization
TSS: Transcriptional start site
uIA: Unruptured intracranial aneurysms

## 1 Introduction

Intracranial aneurysms (IA) represent confined dilation of the intracranial arterial wall. Ongoing studies have identified hypertension, smoking, and female sex as risk factors for IA, while the pathogenesis is hypothesized to involve hemodynamic stress, endothelial and smooth muscle cell defects, myointimal hyperplasia, and inflammation [1]. IA prevalence is estimated at 1%–3% of the population [2]. The progress in diagnostic neuroimaging techniques has led to a rising identification of asymptomatic unruptured intracranial aneurysms (uIA) [3]. Given the devastating consequences following intracranial aneurysm (IA) rupture, a high mortality rate of 35%–65%, and a severe neurological morbidity rate of 30%, prompt medical intervention for uIA is imperative [4]. The decision‐making for prophylactical surgical intervention becomes crucial, as the potential complication risks may outweigh the risk of spontaneous rupture [5]. Even in patients without neurological deficits postintervention, concerns persist regarding recurrence, long‐term antiplatelet therapy, delayed rupture, seizures, and significant costs [6, 7]. Additionally, periodic radiological follow‐ups impose financial and psychological burdens, impacting quality of life [8, 9]. Ideally, pharmaceutical treatment was sought not only to impede aneurysm growth but also to prevent lethal outcomes resulting from the rupture.

Current drug therapies for IA include antihypertensives, lipid‐lowering agents, antihyperglycemics, and nonsteroidal anti‐inflammatory drugs [10, 11]. A cross‐sectional study of 310 aneurysmal subarachnoid hemorrhage (aSAH) and 887 uIA patients found that calcium channel blockers and angiotensin II receptor blockers were inversely associated with aSAH [12]. Atorvastatin use correlated with reduced uIA growth, with fewer cases in users compared to nonusers [13]. Despite ongoing debate, aspirin was suggested to slow uIA growth and reduce aSAH incidence due to its anti‐inflammatory properties [14]. However, these drugs, typically used for cardiovascular disease prevention, introduce potential confounders when coadministered, and their side effects are concerning as they are not IA‐specific [12]. Given the low yield of licensed drugs under the current drug development model, primarily due to late‐stage failures in randomized clinical trials (RCTs), IA‐targeted drug development faces challenges, particularly in the absence of an economically sustainable alternative to RCTs [15].

Mendelian randomization (MR) utilizes allelic randomization during meiosis and irreversible genotype exposure at conception, mimicking an RCT to minimize confounding [16]. It has been effectively employed to identify novel therapeutic targets by inferring causal effects of druggable target genes proxied by nearby expression quantitative trait loci (eQTLs) on disease‐associated genetic variants [17]. Assessing the causal effects of variants linked to eQTLs on IA risk can be analogous to evaluating the efficacy of long‐term medication targeting the same gene, thus enhancing drug development translation rates cost‐effectively [18]. However, previous studies employing MR to identify IA drug targets often lacked causal validation in independent cohorts or did not comprehensively assess pleiotropic effects, which could lead to the identification of seemingly viable drug targets due to unmeasured pleiotropic influences [19, 20].

Hence, the current druggable genome‐wide MR study aimed to identify robust therapeutic targets for IA. It specifically attempted (1) to scan for pharmacological treatment for IA in general; (2) to explore potential drugs mitigating uIA and preventing aSAH. By integrating genomic and phenomic data, the study systematically screened significant and confidence‐inspiring druggable genes through the discovery and validation stages. It also explored the ischemic stroke risks associated with selected druggable genes and assessed potential side effects through phenome‐wide Mendelian randomization (Phe‐MR) analysis. Unmeasured pleiotropy was further adjusted with multivariable MR for additional robustness.

## 2 Methods

### 2.1 Study Design

**FIGURE 1:**
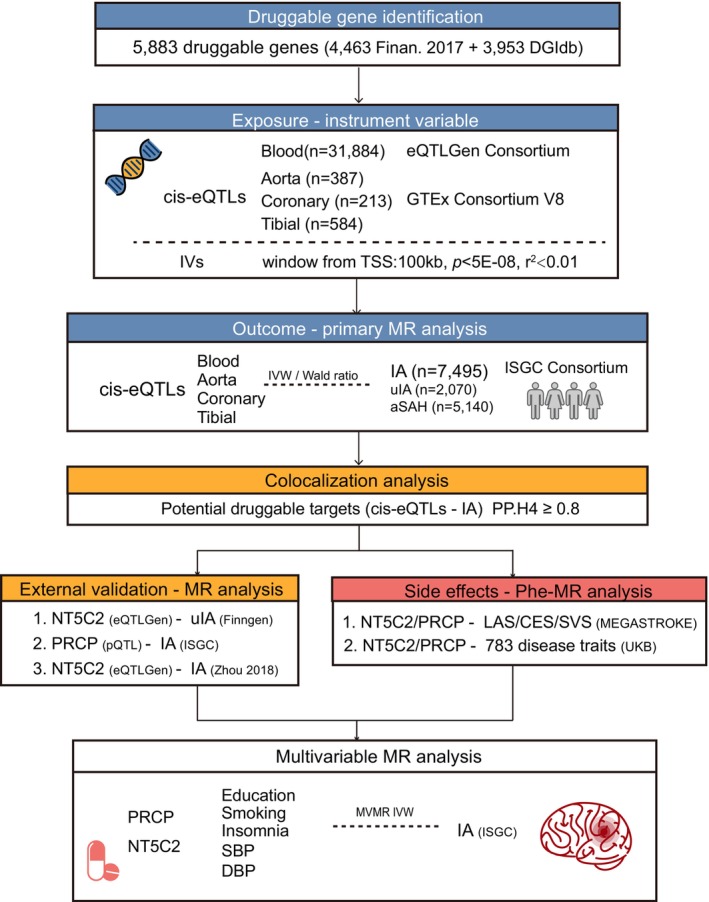
Overview of the study design. aSAH, aneurysmal subarachnoid hemorrhage; DBP, diastolic blood pressure; DGIdb, Drug–Gene Interaction Database; IA, intracranial aneurysm; IV, instrumental variable; TSS, transcriptional start site; IVW, inverse variance weighted MR method; ISGC consortium, International Stroke Genetics Consortium; MEGASTROKE, multiancestry genome‐wide association study of 520,000 subjects identifies 32 loci associated with stroke and stroke subtypes; SBP, systolic blood pressure; uIA, unruptured intracranial aneurysm; UKB, UK biobank.

The present study was designed to identify potential druggable targets for IA with MR (Figure 1). It adhered to the Strengthening the Reporting of Observational Studies in Epidemiology Using Mendelian Randomization (STROBE‐MR) guidelines (Additional Table 3) [21]. The data collection methods and ethical protocol in the original study received approval from the ethics committee of the participating sites, and informed consent was obtained from all participants.

### 2.2 Identification of Druggable Genes

The druggable genome consists of genes encoding proteins that can be targeted by drug‐like small molecules, identified through sequence and structural similarities to known drug targets [22]. A total of 5883 candidate druggable genes with HGNC names were retrieved from Finan's study and the Drug–Gene Interaction Database (DGIdb, https://www.dgidb.org) (Additional Table 1). Finan and colleagues distinguished 4463 druggable genes from over 20,000 genes in humans [22]. Additionally, 3953 genes from DGIdb's druggable categories were included [23].

### 2.3 Exposure Data for Druggable Target Discovery and Validation

*Cis‐*eQTLs for 16,987 genes in blood were sourced from eQTLGen (https://eqtlgen.org/) with data from 31,684 European‐ancestry samples (Figure 1) [24]. Significant *cis‐*eQTLs from arterial tissues (aorta, *n* = 387; coronary artery, *n* = 213; tibial artery, *n* = 584) were obtained from the Genotype‐Tissue Expression (GTEx) project V8 (https://gtexportal.org/) [25]. The GTEx consortium characterizes gene expression using 15,201 RNA‐sequencing samples across 49 tissues from 838 donors. Moreover, plasma *cis‐*pQTLs from Ferkingstad's study and serum *cis‐*pQTLs from Gudjonsson's study were used to validate the genomic target finding [26, 27]. Ferkingstad's study analyzed 4907 plasma proteins with the SomaScan proteomics platform in 35,559 Icelanders, identifying 18,084 correlations between genetic variants and plasma protein levels, adjusted for age and sex. Gudjonsson's study found 4035 independent associations between genetic variants and 2091 serum proteins in 5368 Europeans. Summary statistics of blood pressure, educational attainment, insomnia, and smoking were all assembled for multivariable MR (Table S1) [28, 29].

To generate genetic instrument variables (IVs), eQTLs with *p* values less than 5E‐08 and within a 100 kb window from each gene's transcriptional start site (TSS) were selected. The selected SNPs were then clumped at *r* ^2^ < 0.01 according to the 1000 Genome reference panel [30].

### 2.4 Outcome Data

The primary outcome data were derived from the largest IA GWAS summary‐level data of multiple ancestries by the International Stroke Genetics Consortium (ISGC), including 7495 cases and 71,934 controls. It was divided into uIA (*n* = 2070) and aSAH (*n* = 5140), which were manifested with imaging [31]. To minimize potential bias from population heterogeneity, this study utilized genetic data from European individuals. The IAs of all enrolled patients were the saccular type.

Additional IA summary statistics (uIA and aSAH) with 453,828 were obtained from the FinnGen Consortium R12 release (https://www.finngen.fi/en/access_results) based on Finnish samples as the validation cohort [32]. The uIA cohort included 3310 cases of unruptured cerebral aneurysms, while the aSAH cohort included 6236 cases treated surgically, excluding other cardiovascular diseases. These data were adjusted for sex, age, genotyping batch, and other principal components. A separate validation IA cohort of British ancestry (380 cases and 399,017 controls) by Zhou was also included [33].

Summary statistics of ischemic stroke subtypes, including large artery atherosclerosis (LAS, *n* = 6399), cardioembolic stroke (CES, *n* = 10,804), and small vessel stroke (SVS, *n* = 6811), were retrieved from the GIGASTROKE consortium due to genetic risk factors shared by both hemorrhagic and ischemic stroke subtypes [34]. All the exposure and outcome data are detailed in Table S1.

### 2.5 Mendelian Randomization Analysis

The main Mendelian randomization (MR) method employed was the inverse variance weighted (IVW) method, with the Wald ratio method considered only as an alternative for a single IV. To validate the results obtained through the IVW‐MR method, the weighted median and MR‐Egger analyses were also performed. The weighted median ensures consistent effect estimates if less than half of the genetic instruments are invalid, while MR‐Egger adjusts for pleiotropic effects [35, 36]. The effects of genetic variants were considered statistically significant when the *p* value of IVW‐MR was less than 0.05 and the effect values of various MR methods showed consistently positive or negative.

The “MVMR” package was used for multivariable MR analysis to adjust *NT5C2* and *PRCP* for potential confounders, including modifiable IA risk factors such as blood pressure, educational attainment, insomnia, and smoking [37, 38]. Under instrumental variable assumptions, the instruments for multivariable MR were constructed by integrating shared IVs for each exposure at *p* < 5E‐08 and clumping with an *r* ^2^ < 0.1 within 10,000 kb. The multivariable IVW method was employed as the primary approach [39]. Multivariable MR–Egger and the MR–Lasso methods were also applied to adjust for both measured and unmeasured pleiotropy [40]. Heterogeneity and pleiotropy were assessed using Cochran's Q statistic and the MR‐Egger intercept test [41].

### 2.6 Sensitivity Analysis

The instrument SNPs were kept if the *F*‐statistic value was over 10. Cochran's Q statistics were used to reveal the heterogeneity where *p* < 0.05 [42]. The horizontal pleiotropy was indicated when *p* < 0.05 by using Mendelian Randomization Pleiotropy RESidual Sum and Outlier (MR‐PRESSO) analysis and MR Egger intercept [43]. The SNP outliers were excluded with a leave‐one‐out analysis.

### 2.7 Bayesian Colocalization Analysis

Bayesian colocalization analysis was performed using the R package COLOC (version 5.2.2) to identify shared causal genetic variants between eQTLs and IA outcomes within a 100 kb window of each gene's transcription start site, considering that a SNP may be associated with multiple genes [44]. A prior probability of 1E‐04 was set for both gene expression (*p*_1_ = 1E‐04) and IA outcomes (*p*_2_ = 1E‐04), with a prior probability of 1E‐05 (*p*_12_ = 1E‐05) for a single variant associated with both traits, indicating the significance of association between an SNP and the tested gene, IA outcomes, or both. The analysis provided posterior probabilities (PP) for five hypotheses: PPH0 (SNPs not associated with either trait), PPH1 (SNPs associated with gene expression only), PPH2 (SNPs associated with IA risk only), PPH3 (distinct variants associated with IA risk and gene expression), and PPH4 (a shared SNP associated with both traits). Variants were defined as colocalization with evidence when the PPH4 was no less than 0.8 (PPH4 ≥ 0.8).

### 2.8 Phenome‐Wide MR Analysis

The summary statistics of disease‐associated SNPs including 1403 traits from the UK Biobank cohort (*n* ≤ 408,961) were obtained from the Scalable and Accurate Implementation of Generalized Mixed Model (SAIGE V.0.29) GWAS (https://www.leelabsg.org/resources) to address imbalanced case–control ratios. A total of 783 traits with cases over 500 were retained due to statistical power (Additional Table 2). MR analyses were then performed for causal inference between the druggable genes and the included traits via the IVW method.

### 2.9 Statistical Analysis

All the statistical analyses were performed in RStudio (version 1.1.463, R version 4.3.1) with the packages “TwoSampleMR” and “MR‐PRESSO.” *p* values underwent false discovery rate (FDR) adjustment (Benjamini–Hochberg method) for multiple testing, with values less than 5% considered statistically significant. In the validation stage, nominal *p* values less than 0.05 were considered statistically significant.

## 3 Results

### 3.1 Identification of Druggable Genes for IA

**FIGURE 2:**
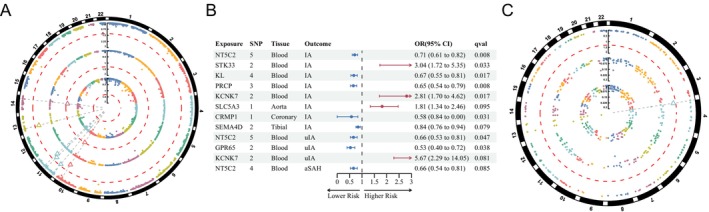
MR analysis with *cis‐*eQTLs of blood and artery in the discovery stage. (A) Circos Manhattan plot illustrates the significant druggable genes (triangle) on IA (outer circle), uIA (middle circle), and aSAH (inner circle) in blood with *cis‐*eQTLs (dots) from the eQTLGen consortium across the entire genome (outermost black circle); the y‐axis indicates the value of −log_10_(*q*) and the red dashed line indicates the *p‐FDR* value of 0.05. (B) Forest plot presents the causal effects of the significant (and most nearly significant) druggable genes in blood and arterial tissue on aneurysmal outcomes. (C) Circos Manhattan plot for *cis‐*eQTLs of *CRMP1* (triangle) significantly associated with IA (outer circle); the y‐axis indicates values of −log_10_(*q*) and the red dashed line indicates a *p‐FDR* value of 0.05.

In the discovery stage, significant *cis‐*eQTLs (*p* < 5E‐08) for 3458 druggable genes were identified within a 100 kb window from the TSS in blood, sourced from the eQTLGen consortium. Five gene targets were significantly associated with IA risks using the IVW method (*p‐*_*FDR*_ < 0.05) (Figure 2A). The risk of IA significantly decreased by 30% with increased *NT5C2* expression (OR, 0.70; 95% CI, 0.606–0.823; *p‐*_*FDR*_ = 0.008) (Figure 2B and Table S2). The expression levels of *PRCP* were also significantly associated with a reduction in the IA risk (*PRCP*, OR, 0.65; 95% CI, 0.534–0.810; *p‐*_*FDR*_ = 0.008). No significant heterogeneity, horizontal pleiotropy, or outlier‐driven associations were detected (Table S3).

Due to the lack of intracranial arterial eQTLs, extracranial tissues were analyzed. Aorta artery *cis‐*eQTLs of 1371 druggable genes, coronary artery *cis‐*eQTLs including 486 druggable genes, and tibial artery *cis‐*eQTLs of 1767 druggable genes were obtained from the GTEx for MR analysis (*p* < 5E‐08). Only *CRMP1* in the coronary artery showed an inverse association with IA risks (OR, 0.69; 95% CI, 0.575–0.835; *p‐*_*FDR*_ = 0.031) (Figure 2B,C).

### 3.2 Identification of Druggable Genes in uIA and aSAH

The druggable target exploration was continued in the IA subtype cohorts, including the uIA and aSAH cohorts. The *NT5C2* expression in the blood also showed an inverse association with uIA risk (*NT5C2*, OR, 0.66; 95% CI, 0.533–0.808; *p‐*_*FDR*_ = 0.047) (Figure 2B). No significance was observed regarding heterogeneity and pleiotropy for *NT5C2* expression (Table S4). There were no significant associations between the eQTLs of arterial tissue and uIA risks (Tables S5–S7).

However, few druggable genes were associated with aSAH. The causal association between blood *NT5C2* expression and the risk of aSAH was not observed, despite its proximity to statistical significance (*p‐*_*FDR*_ = 0.085) (Figure 2B). It exhibited no significant causal effects of arterial genes on aSAH risk associations (Tables S8–S10).

### 3.3 Colocalization Analysis and External Validation

**FIGURE 3:**
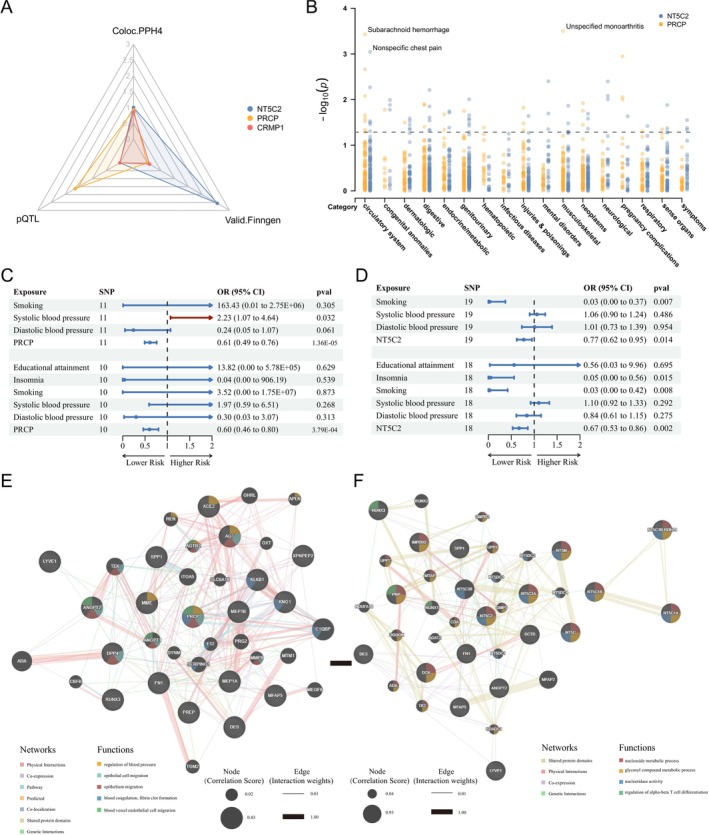
Follow‐up analysis. (A) Radar plot showing potential druggable genes, *NT5C2*, *PRCP*, and *CRMP1* for IA showing the value of −log_10_(*p*) in MR analysis with *cis‐*pQTL and IA cohort in the Finngen study as validation (valid.Finngen), while values of posterior probability H4 (PPH4) were kept the same. (B) Manhattan plot for phenome‐wide MR results of *NT5C2* and *PRCP* with 783 disease traits from UKB; a dot represents a disease trait; the y‐axis indicates the value of −log10(q) and the black dashed line indicates the *p* value of 0.05. (C) Forest plot for the multivariable MR analysis incorporating *PRCP*, smoking, systolic blood pressure (SBP), diastolic blood pressure (DBP) with IVW method as a smoking‐SBP‐DBP‐*PRCP* model; and multivariable MR including all modifiable risk factors and *PRCP* as the *PRCP*‐all model. (D) Forest plot for the multivariable MR analysis incorporating *NT5C2*, smoking, SBP, and DBP with IVW method; and multivariable MR with all modifiable risk factors. (E) Protein–protein interaction network with IA characteristic genes for PRCP. (F) Protein–protein interaction network with IA characteristic genes for NT5C2.

A colocalization study was then performed to determine whether the significant druggable eQTLs shared causal genetic variants with IA outcomes. The *cis*‐eQTLs of *NT5C2* in blood shared a common causal SNP with IA (PPH4 = 0.999), and *PRCP* exhibited a shared causal SNP with IA (PPH4 = 0.921), as well as *CRMP1* (PPH4 = 0.853) (Figures 3A, S1 and Table S11). Therefore, *NT5C2*, *PRCP*, and *CRMP1* were identified as potential druggable genes for IA based on the significant causal effects and the common causal variants.

The identified druggable genes were subsequently validated with the cerebral aneurysm GWAS from the FinnGen cohort and the separate IA cohort by Zhou in 2018 [33]. The causal effect of *NT5C2* expression on the uIA risk was replicated, showing an OR of 0.81 in the FinnGen cohort (95% CI, 0.707–0.930; *p* = 0.003) (Figure 3A and Table S12). However, the effects of *PRCP* on uIA and aSAH risks were insignificant in the FinnGen cohort, possibly requiring further validation with IA statistics (Table S12). In the validation with Zhou's IA cohort, the inverse association between *NT5C2* and IA risk was confirmed with an OR of 0.68 (95% CI, 0.469–0.983; *p* = 0.041), while no causal effect was observed for *PRCP* (Table S13). *CRMP1* could not be validated due to insufficient IVs in either cohort.

In addition to gene expression level, the plasma and serum *cis‐*pQTLs of druggable genes were investigated with IA risk. It revealed that a genetically predicted increase in the plasma protein level of *PRCP* reduced IA risk with an OR of 0.734 (95% CI, 0.561–0.959; *p* = 0.023) (Table S14). The serum protein level of *PRCP* was also inversely associated with IA (OR, 0.72; 95% CI, 0.542–0.956; *p* = 0.023) (Table S14). *CRMP1* and *NT5C2* were not validated at the protein level.

### 3.4 Side Effects Evaluation for IA Druggable Genes

Considering that common genetic risk factors were shared by both hemorrhagic and ischemic stroke, inferring the effect of putative druggable targets on ischemic stroke was conducted with MR analysis [45]. However, causal links with LAS, CES, or SVS were not observed for *PRCP* (Table S15). Similarly, *NT5C2* showed no significant associations with ischemic stroke subtypes (Table S15).

Beyond ischemic stroke, the side effects of *NT5C2 and PRCP* were assessed across 783 disease traits using Phe‐MR analysis for more comprehension. Neither *PRCP* nor *NT5C2* displayed statistically significant associations with any of the traits tested, including correlation with elevated blood pressure without a diagnosis of hypertension, hypertension, essential hypertension, and hypertension complicating pregnancy, childbirth, and the puerperium. No statistically significant side effects were observed for *NT5C2* or *PRCP* in the Phe‐MR (Figure 3B). However, some disease traits showed nominal significance regarding their association with *NT5C2* and *PRCP* (Tables S16 and S17).

Given the potential involvement of blood pressure and other modifiable IA risk factors such as educational attainment, insomnia, and smoking, multivariable MR analysis was conducted to adjust the direct effects of *NT5C2* and *PRCP* on IA [37, 38]. Considering the significant effect of smoking on IA in the univariable MR, it first showed that *PRCP* remained associated with IA even after adjustments for smoking, systolic blood pressure (SBP), and diastolic blood pressure (DBP) (*PRCP*, OR, 0.61; 95% CI, 0.487–0.761; *p* = 1.36E‐05) (Figure 3C, Tables S18 and S19). There was no evidence of heterogeneity or horizontal pleiotropy in the smoking‐SBP‐DBP‐*PRCP* model (Table S20). Further adjustments for all modifiable risk factors confirmed the direct effect of *PRCP* on IA (OR, 0.60, 95% CI, 0.457–0.797; *p* = 3.79E‐04), with no significant heterogeneity or pleiotropy (Figure 3C and Table S21).

*NT5C2* also demonstrated a causal relationship with IA, independent of smoking and blood pressure (*NT5C2*, OR, 0.77; 95% CI, 0.623–0.949; *p* = 0.014) (Figure 3D). This significant causal effect persisted in the *NT5C2*‐all modifiable risk factors model (*NT5C2*, OR, 0.67; 95% CI, 0.527–0.860; *p* = 0.002), with no significant heterogeneity or pleiotropy observed (Figure 3D and Table S21).

To explore the potential molecular mechanism of the target genes in IA, protein–protein interaction (PPI) network analysis was conducted using GeneMANIA (https://genemania.org/) [46]. Integrating 23 characteristic IA genes identified through single‐cell RNA sequencing, the PPI networks for PRCP and NT5C2 were mapped along with their interacting proteins. Beyond regulating blood pressure via the AGT pathway, PRCP might influence IA through the PRCP‐DPP4‐ANGPT2 axis, modulating endothelial proliferation (Figure 3E). NT5C2 could affect IA indirectly by interfering with T‐cell differentiation via PNP and RUNX1, potentially involving endothelium modulation through ANGPT2 (Figure 3F).

## 4 Discussion

This study identified *NT5C2* and *PRCP* as potential drug targets for IA through a druggable genome‐wide MR analysis. The discovery‐validation strategy and colocalization analysis helped to narrow down five candidate genes. Notably, *NT5C2* and *PRCP* did not show significant side effects across 783 evaluated disease traits, including ischemic stroke and elevated blood pressure. They were significantly associated with IA even after adjusting for the known modifiable risk factors.

The primary MR analysis using *cis‐*eQTLs from blood samples revealed five candidates. The *cis‐*focused approach proved less likely to violate the horizontal pleiotropy assumption compared to *trans* instruments located in other gene regions [47]. Given IA's potential origins in endothelial impairment and vessel wall inflammation, *cis‐*eQTLs from arterial tissues were also analyzed [48]. However, few significant targets aligned with druggable genes in arterial tissues, likely due to the small sample size and limited probes in the GTEx. Intracranial arteries, distinct from extracranial arteries due to the absence of an elastic fiber layer, might not be fully represented by arterial tissue data [49].

*NT5C2* encodes a highly conserved enzyme, cytosolic 5'‐nucleotidase II (cN‐II), involved in purine nucleotide metabolism and phosphate transferase activity [50]. It has been extensively studied in cancers, particularly leukemia [51]. In this study, genetically determined *NT5C2* levels in blood were inversely associated with IA, uIA in particular, suggesting a protective role and a therapeutic pathway for reducing IA formation and impeding uIA progression. Colocalization analysis and external validation with the Finngen cohort reinforced the causal association between *NT5C2* and uIA. In addition to its involvement in blood pressure regulation, previous trans‐ancestry GWAS findings, which linked *NT5C2* to brain arterial diameter, further support its role in IA formation [52].

Prolylcarboxypeptidase (PRCP) is a lysosomal serine protease that cleaves peptide substrates when the penultimate amino acid is proline [53]. It acts through three main pathways: the pro‐opiomelanocortin system for energy metabolism, the renin‐angiotensin system for vascular homeostasis, and the kallikrein‐kinin system for inflammation [53]. The *PRCP* ^gt/gt^ mice exhibited elevated blood pressure, higher thrombosis risk, and reduced angiogenesis, highlighting its role in vascular health [54]. The current study exposed a protective role of blood *PRCP* in IA supported by eQTL, pQTL, and colocalization evidence, consistent with a previous report that excluded sex chromosome candidates but overlooked sex hormones as IA risk factors [20]. The slight discrepancy of insignificance for the causal effect of *PRCP* on aSAH might come from differed linkage disequilibrium settings, with the same linkage disequilibrium threshold *r* ^2^ < 0.1 repeating the significant association for *PRCP* and aSAH in the prior study (Table S22) [20]. Considering the pathogenesis of IA, characterized by endothelial dysfunction, neointimal hyperplasia, and thrombosis, *PRCP* might reduce the risk of IA formation by promoting endothelium motility and survival under hemodynamic stress and maintaining endothelialization to prevent smooth muscle cell proliferation and neointimal thickening [55].

The Phe‐MR analysis revealed few disease traits associated with *NT5C2* and *PRCP* exposure, including ischemic stroke subtypes, while excluding typical side effects related to *NT5C2*, such as leukemia and psychiatric disorders [56]. It also ruled out the potential pleiotropy for *PRCP*, which was involved with hypertension‐related traits, cardiovascular and renal disorders, coagulation defects, and metabolic disorders like obesity [57]. The weak associations between these druggable target genes and the disease traits examined, including the ischemic stroke subtypes, suggested that targeting these genes was relatively safe, though further clinical studies were warranted. Additionally, the direct effects of the target genes were evaluated through the multivariable MR.

The multivariable MR analysis confirmed the independent causal effects of *NT5C2* and *PRCP* on IA, even when accounting for established modifiable risk factors such as blood pressure, smoking, insomnia, and educational attainment. Both *NT5C2* and *PRCP* were involved in blood pressure regulation, with the PRCP polymorphism (E112D) associated with hypertension and NT5C2 correlated with elevated blood pressure [58]. *NT5C2* was implicated in smoking initiation, suggesting potential mediation of IA risk via nicotine exposure [59]. The results indicated that *PRCP* and *NT5C2* might play crucial roles in IA formation, even in cases of aSAH, when compared to SBP, smoking, and insomnia [38]. Therefore, increasing *NT5C2* and *PRCP* expression could prove effective in treating IA, potentially halting the progression of unruptured IA and preventing aSAH. The independence also suggested that the genes reduced IA risk through alternative pathways, such as the PRCP‐DPP4‐ANGPT2 and NT5C2‐ANGPT2 pathways identified in the PPI network analysis [46]. PRCP might additionally interfere with endothelium function by regulating reactive oxygen species, while NT5C2 probably influenced IA risk through circulating T cell manipulation [54]. However, further molecular investigations were needed to specify the mechanisms.

The current research marked the first attempt to identify drug targets for IA with druggable genome‐wide MR analysis incorporating large‐scale genomic, proteomic, and phenomic data. Firstly, the genetic evidence enhanced the prioritization of drug targets for advancement into clinical trials [18]. Secondly, its colocalization analysis and discovery‐validation strategy ensured the robustness of identified target genes. In contrast to previous MR studies on IA drug targets, which relied on transcriptome‐wide association studies (TWAS) to establish associations rather than causality and overlooked potential pleiotropy, our approach ensured causal validation [19]. Additionally, the safety and alternative indications for the targets were systematically evaluated using the SAIGE‐balanced disease traits through Phe‐MR analysis. The significant causal effects of the identified druggable genes remained robust even after adjusting for all known modifiable IA risk factors. These genes provided a promising direction for future drug development in IA, offering time‐efficient and cost‐effective pharmaceutical alternatives that could reduce the need for surgical interventions and provide nonsurgical options for asymptomatic unruptured IA patients deliberating surgery. By targeting these genes, effective treatments could minimize surgical risks while being financially beneficial.

Several limitations in this study warranted cautious interpretation of the results. Firstly, the small number of IVs aligned with genes of the druggable genome resulted in a limited number of identified drug targets. The restricted genetic proxy SNPs for each target gene necessitated caution in interpreting the results due to the constrained confidence level. Secondly, the findings were not validated at both the genetic and protein levels simultaneously, underscoring the need for large‐scale validation and further studies on underlying posttranscriptional modifications. The lack of verification at the expression level for *PRCP* could stem from the absence of IA cohorts with a sample size comparable to the ISGC dataset, while the limited IVs after filtering contributed to the lack of protein‐level validation for *NT5C2*.

Thirdly, the study was confined to individuals of European ancestry, limiting the generalization of findings to other ethnic groups. Fourthly, while side effects were analyzed with the Phe‐MR analysis, a more comprehensive understanding of off‐target effects would require future research. Although the robustness of the druggable genes was demonstrated in the presence of all modifiable IA risk factors, directional pleiotropy might not be entirely excluded.

Fifthly, the MR approach in this study assumed a linear relationship between low‐dose drug exposure and outcomes, which might not accurately reflect the effects observed in high‐dose clinical trials over short periods. Lastly, the identified druggable genes, *NT5C2* and *PRCP*, warranted further basic experiments and clinical studies to elucidate their specific mechanisms and actual effects. As MR studies assessed the impact of genetically determined lifelong exposure, the effect size of perturbation against the exposure could be overestimated in short‐term randomized trials [60].

## 5 Conclusion

Overall, this druggable genome‐wide MR analysis identified *NT5C2* and *PRCP* in blood as novel drug targets for IA. *NT5C2* and *PRCP* might reduce the risk of IA in general, and *NT5C2* was found to particularly reduce the risk of uIA or, in part, halt the growth of uIA. The causal effects of *NT5C2* and *PRCP* on IA were also independent of the known IA modifiable risk factors. Targeting *NT5C2* and *PRCP* appeared safe and exclusively effective for IA, including ischemic stroke and hypertension. These genes offered a promising pathway for future IA drug development, which could lessen the reliance on surgical interventions and provide nonsurgical alternatives for asymptomatic unruptured IA patients who might otherwise require invasive procedures.

## Author Contributions

Hong‐Qi Zhang and Yu‐Xiang Fan. conceptualized the design of the study. Yu‐Xiang Fan, Di Lu, Cheng‐Bin Yang, Zi‐Hao Song, and Jing‐Wei Li acquired and analyzed data. Yu‐Xiang Fan drafted the initial manuscript. Yu‐Xiang Fan, Di Lu, Yi‐Guang Chen, and Yong‐Jie Ma prepared figures. Hong‐Qi Zhang, Jing‐Wei Li, and Yong‐Jie Ma reviewed and edited the manuscript. All authors have read and approved the manuscript.

## Ethics Statement

The authors have nothing to report.

## Consent

The authors have nothing to report.

## Conflicts of Interest

The authors declare no conflicts of interest.

## Supporting information


**Figure S1.** Colocalization plots between (A) PRCP; (B) NT5C2; (C) CRMP1 and IA.

**Table S1.** Sources of GWAS summary statistics.
**Table S2**. Summary statistics of cis‐eQTL of NT5C2 and PRCP and IA in the primary MR analysis.
**Table S3**. Sensitivity analysis of causal associations between eQTL‐eQTLGen and IA risks.
**Table S4**. Sensitivity analysis of causal associations between eQTL‐eQTLGen and uIA risks.
**Table S5**. Causal associations between eQTL‐GTEx in aorta artery and uIA risks.
**Table S6**. Causal associations between eQTL‐GTEx in coronary artery and uIA risks.
**Table S7**. Causal associations between eQTL‐GTEx in tibial artery and uIA risks.
**Table S8**. Causal associations between eQTL‐GTEx in aorta artery and aSAH risks.
**Table S9**. Causal associations between eQTL‐GTEx in coronary and aSAH risks.
**Table S10**. Causal associations between eQTL‐GTEx in tibial artery and aSAH risks.
**Table S11**. Colocalization analysis of potential drug target genes associated with IA.
**Table S12**. Validation for druggable cis‐eQTLs in the Finngen cohort.
**Table S13**. Additional validation for druggable cis‐eQTLs in the IA cohort by Zhou.
**Table S14**. Validation for causal associations between pQTLs in blood and IA risks.
**Table S15**. Validation for causal associations between drug targets and ischemic stroke risks.
**Table S16**. Phe‐MR analysis of NT5C2 with 783 disease traits of nominal significance.
**Table S17**. Phe‐MR analysis of PRCP with 783 disease traits of nominal significance.
**Table S18**. Validation for casual associations between modifiable risk factors and IA.
**Table S19**. Sensitivity analysis for univariable MR of modifiable risk factors.
**Table S20**. Multivariable MR for PRCP using MR–Egger and MR‐Lasso.
**Table S21**. Sensitivity analysis of multivariable MR for PRCP and NT5C2 adjustments.
**Table S22**. Sensitivity analysis of causal effect of PRCP on aSAH under differed linkage disequilibrium thresholds.

**Appendix S1.** Additional Table 1. Candidate druggable genes.
Additional Table 2. 783 disease traits in the SAIGE UKBiobank dataset.
Additional Table 3. The STROBE‐MR checklist.

## Data Availability

All data included in the current research are publicly available. Sources of summary statistics are elaborated in Additional Table 1.
