# Supplementary figures and images for: Multiomic Underpinnings of Drug Targets for Intracranial Aneurysm: Evidence From Diversified Mendelian Randomization
Source: CNS Neurosci Ther. 2025 May 10;31(5):e70430. doi: 10.1111/cns.70430 (PMC12064948; doi:10.1111/cns.70430)

A

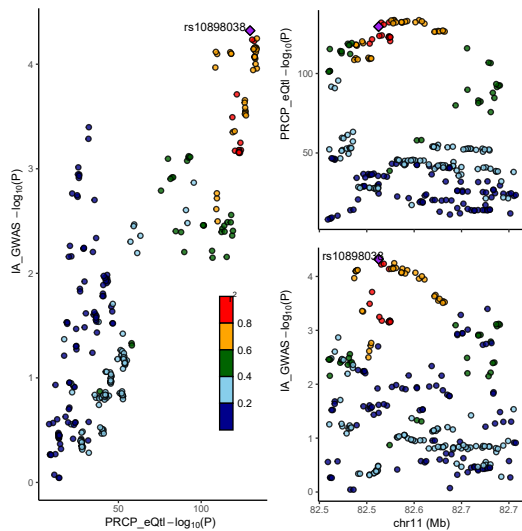

B

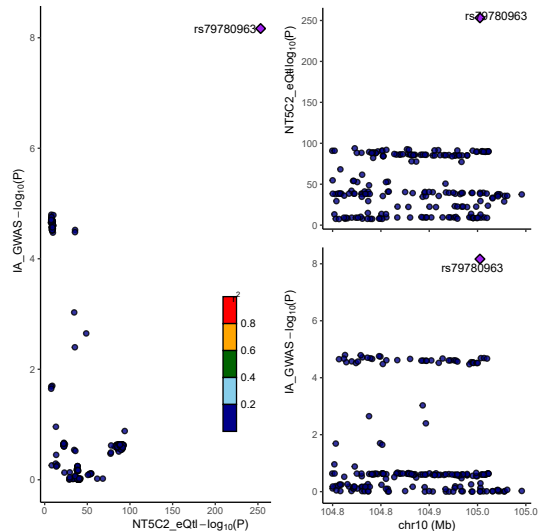

C

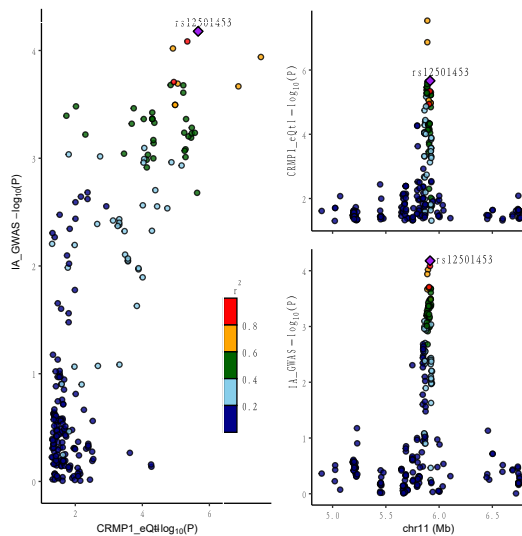

Supplement: Supplementary file 1 — Figure S1. Colocalization plots between (A) PRCP; (B) NT5C2; (C) CRMP1 and IA. [file CNS-31-e70430-s002.pdf]
